# “Cooperative Learning Does Not Work for Me”: Analysis of Its Implementation in Future Physical Education Teachers

**DOI:** 10.3389/fpsyg.2020.01539

**Published:** 2020-07-02

**Authors:** David Hortigüela-Alcalá, Alejandra Hernando-Garijo, Sixto González-Víllora, Juan Carlos Pastor-Vicedo, Antonio Baena-Extremera

**Affiliations:** ^1^Faculty of Education, University of Burgos, Burgos, Spain; ^2^Faculty of Education of Cuenca, University of Castilla-La Mancha, Cuenca, Spain; ^3^Faculty of Education of Toledo, University of Castilla-La Mancha, Toledo, Spain; ^4^Faculty of Education of Granada, University of Granada, Granada, Spain

**Keywords:** physical education, cooperative learning, pedagogical model, models-based practice, initial teacher training

## Abstract

Cooperative learning (CL) is one of the pedagogical models that has had more application in the area of Physical Education (PE), being highly worked in the initial training of teachers. The aim of the study is to check to what extent future PE teachers are able to apply in the classroom the PE training they have received at university, deepening their fears, insecurities and problems when carrying it out. Thirteen future PE teachers (7 girls and 6 boys) aged 20.87 ± 1.43 participated and, after having been trained in CL in various subjects, applied it in the classroom during their internship. They were selected through purposeful non-probability sampling. A qualitative methodology was used, being the interviews, the teaching diaries and the seminars with the tutor the instruments of data collection used. Three categories of analysis were used: (a) initial expectations in the application of the CL; (b) problems encountered in its implementation; (c) reflection about its application in the future. The results showed how the future teachers did not see their expectations of success fulfilled, encountering resistance from both students and teachers in PE. Furthermore, they reflect the need to continue training in a model that has infinite nuances so that it can be implemented satisfactorily. It is necessary to continue researching a pedagogical model with so many possibilities in the area of PE and with so much transversality on a social level.

## Introduction

Society is constantly evolving, transforming different implicit realities under concrete socio-cultural factors (Sudakova and Astafyeva, 2019). In the face of this, the school cannot remain in the background, and concrete mechanisms of permanent formation must be established that are in line with the current situation. This connection between society and school must be closely linked to the role played by the university, intentionally addressing research-action processes that favor and promote true educational innovation (Roffeei et al., 2018). If continuing education is especially relevant to educational advancement, initial training takes on special significance, due to its great impact on the creation of the professional identity of teachers (Androusou and Tsafos, 2018). In this sense, without a doubt, one of the greatest challenges lies in seeing how this university training can be successfully replicated by future teachers in schools. It is also necessary to focus on other factors that may be important in the development of a teacher’s identity, such as the transferability of learning, academic motivation or the teacher’s perception of competence.

This relevance of initial teacher training takes on an even greater dimension when it comes to PE, where the use of corporeality is present. The body is an indissoluble element of the individual, and for this reason, from the PE it must be worked on with clear pedagogical purposes that go beyond biomedical and physiological parameters (Kennedy et al., 2019). The acquisition of values in the practice of physical activity is not achieved by the mere fact of doing it, but rather there must be an ethical base built under very clear and defined social purposes (Schenker, 2019). This is where pedagogical models of teaching come into play, especially that of CL, which has already demonstrated benefits in the relational, motivational and learning environments of students (Casey and MacPhail, 2018). Applying the CL model in PE with guarantees ensures a climate of respect, empathy and initial solidarity on which to base motor practice, something that favors the self-concept and autonomy of students when carrying out their daily activities in the classroom. This relates to the theory of self-determination, demonstrating how a good social climate in the group affects the intrinsic motivation of students, the identified regulation and task orientation (Sevil et al., 2016). Starting from a climate of equality in the PE classroom is essential, since corporal exposure to others is constant, which on many occasions generates fears and insecurities in the face of failure and the consequent mockery of one’s classmates (Duncan and Bellar, 2015). This has a direct consequence in society, where on many occasions the criteria of honesty and nobility have been lost, with the ego taking precedence and the search for individual benefit over the common good.

However, applying the CL model in PE effectively is not easy, since it requires a high level of mastery in the knowledge of its implementation phases and the elements that constitute it. In certain cases, faculty believe that they are applying it adequately, when in reality what they are doing are only isolated activities of a cooperative nature that are not integrated into a specific methodology (Morgan, 2019). And if it is not easy to apply it consistently, it is even more complex to teach it with guarantees of replicability in the classroom. On various occasions, the initial training of teachers, due to the idyllic context in which the teaching takes place, can lead to a feeling of control over the learning acquired by the student, something that is not always corroborated when he or she tries to apply it in the classroom (Jones, 2019). Studies carried out with future PE teachers, with a pre-test design, showed that the CL is fundamental to apply it as a scaffolding in the teaching process. Specifically, this scaffolding favors variables such as practical knowledge, teaching skills and self-efficacy (Legrain et al., 2019). However, it was shown that when this scaffolding is not structured and applied in a transversal way in the training, problems related to teaching skills appear. Research has shown the difficulties PE teachers have in introducing new technologies into cooperative learning (CL) when they do not intentionally use them throughout the course (Bodsworth and Goodyear, 2017). Sometimes PE teachers confuse the implementation of CL with the implementation of specific activities and games, which causes problems for the class to act truly as a group (Casey et al., 2015). Another of the main problems of teachers in the application of the CL is found in the difficulties that students have in solving their problems, so it is necessary to establish longitudinal programs of intervention (Gorucu, 2016). On some occasions, in the teacher’s eagerness to apply the CL in the classroom, they forget what the didactic purposes of the proposed interventions are (Wallhead and Dyson, 2017). Another major problem is generated around the power relationships established in the classroom. The qualitative research of Barker and Quennerstedt (2017), through the work of dance in PE, showed how CL can favor the group climate over the students’ motor skills. Another of the most common reticence among PE teachers to apply CL is the reduction of motor commitment it entails in the classroom. However, research such as that of Altinkok (2017), applied with 6–7 year-old students, in 12 weeks and with a pre-test, showed a greater development of motor skills in the experimental group.

Therefore, and considering the different social benefits that the implementation of the CL in the school PE has demonstrated, it is necessary to verify the effects of replicability that its teaching has on the future PE teachers. Therefore, the objective of the study is to analyze to what extent future PE teachers are able to apply in the classroom the training on CL that they have received at the university, deepening in their fears, insecurities and problems when carrying it out. This is a significant contribution to the existing literature on the subject, since, in addition to generating new evidence on the applicability of this model, it uses a contrast of qualitative instruments that allow for reflection and redirection of the practices of teachers throughout their process of implementation in the classroom.

### Cooperative Learning as an Essential Element in the Initial Training of Physical Education Teachers

In the initial training of PE teachers, in addition to the specific contents of the discipline, it is necessary to intentionally address a diversity of methodological aspects that are transferable to the classroom in which they carry out their professional activity (Capel et al., 2018). These are related to the design of classes, the organization of groupings, time management or the application of assessment. All of them are clearly immersed in the development of pedagogical models, within which the CL is essential for several reasons. It starts from an important social base, which makes dialogue and consensus building a priority in its implementation. In addition, and starting from the empowerment of the capacities of the group members, the achievement of challenges is sought jointly, which favors the feeling of affiliation (Hortigüela-Alcalá et al., 2019). This process must be articulated under the promotion of the generation of positive motor experiences, proposing activities where the level of achievement is perceived as affordable and motivating by the students (Koka, 2013). All the typology of contents that includes the subject of PE has a place in the CL, always being implicit the values of solidarity, empathy and respect for others. These fundamental premises of action in the classroom have a clear connection with the behavioral patterns of today’s society, which allows the establishment of a binomial with the school and with the connection of its learning. Furthermore, it can be applied in a transversal way to other curricular areas, establishing common action protocols regarding student responsibility and autonomy.

Another of the fundamental aspects that justify the teaching of the CL in the initial training of teachers is the increase of the motor possibilities that it entails in the student, since it does not reduce the learning objectives to the achievement of a determined motor performance in an isolated test. Its vision and application is carried out in its widest spectrum, being applicable in diversity of contents, tasks and contexts (Casey and Goodyear, 2015). In addition, it allows the development of emotional bonds between the members of the class, putting themselves in the place of others when it comes to making decisions. This causes that as future PE teachers they rethink how to promote social interactions in the classroom in a positive way, improving the group climate, self-esteem and self-concept of the students. Under this prism of pedagogical action, one of the main purposes of school PE is achieved, which is the generation of positive learning experiences around motor skills. This is connected to what has been called transcontextual analysis, defined as the impact that PE classes have on students so that they later decide to practice physical activity autonomously in their free time (Hagger and Chatzisarantis, 2016). The CL model is also very interesting to be applied from the sports teaching, since it teaches that everyone has to play with everyone in different conditions and situations, going far beyond the mere competition where someone is always defeated (Kolovelonis, 2019). It establishes procedures for playful performance in which everyone has the right and opportunity to win, and the fact of not winning does not mean that someone is eliminated. For this purpose, techniques such as 1-2-4, numbered heads together, Aronson’s puzzle or the collective score are applied.

It is necessary to emphasize from the initial training of the teaching staff that the CL is not only applied in a punctual way through isolated activities, since the key to the success of its implementation is found in delimiting temporary phases in the medium term that allow the class to act in a cohesive way around motor skills (Lafont et al., 2016). This pedagogical awareness leads the future PE teacher to create a professional identity of a cooperative nature, generating an idiosyncrasy and a behavior that defines him/her as a teacher. This has repercussions on such relevant aspects as the selection of content to be developed in the classroom, time management in tasks, the way of carrying out groupings or the proposal and typology of the approach to objectives. Therefore, the transversality that characterizes the CL is usually well accepted among future PE teachers, since their previous experiences do not limit or bias their applicability in the classroom (Yuksel et al., 2019). The design, preparation and evaluation of activities are essential for PE teacher to gain confidence in their professional practice. To this end, it is essential that, in addition to knowing about the diversity of activities, they are able to structure them within a methodology that can be adapted in a variety of contexts (Lafont et al., 2016). However, a novice teacher’s professional beginnings are not easy. Considering the level of teaching experience, it can be seen that new teachers are excessively dependent on the curriculum. Therefore, it is necessary that initial training of PE teachers include more support from experienced teachers in the subject (Viciana and Mayorga-Vega, 2017). While teachers say they are confident in aspects related to health and fitness, they are more hesitant in those related to assessment and classroom control (Randall, 2020). Another of the training deficiencies that future PE teachers admit to have is that related to Special Educational Needs. This subsequently generates problems in the actual treatment of inclusion in the classroom (Maher and Fitzgerald, 2020). It is necessary to work from the initial training of teachers to question the hegemonic practices associated with PE, establishing pedagogical lines that can respond to the needs of a new type of physical education (PE) teacher (Wrench and Garrett, 2015).

All of the above-mentioned benefits are key to the initial training of teachers, thus increasing the guarantees of implementing it in their professional future and thus achieving educational and social transformation. And this is precisely what this study aims to prove, analyzing the effectiveness of its application in the specific school context by new teachers.

## Materials and Methods

### Participants

Thirteen future PE teachers (7 girls and 6 boys) aged 20.87 ± 1.43 participated. This number of participants is ideal in relation to the type of study, instruments and analysis used. All of them had received CL training throughout their university career. This qualification is the Degree in Primary Education, with the speciality in CL, given at the University of Burgos (Spain). There are 5 subjects (30 ECTS or 750–900 h the student spends on the study activity) that make up the specialty, taken in the third and fourth years. Specifically, in the third year there is a subject called pedagogical models in PE, whose main objectives revolve around the teaching of the school PE, with the teaching of the CL model having great weight. It is in this subject that future PE teachers were specifically trained in the application of the CL in the classroom. The model was approached in a theoretical-practical way, analyzing each of its elements and phases and applying them later in the classroom through a diversity of contents. A model of formative assessment and feedback was established in each of the classes, influencing in a reflexive way their learning possibilities. However, in previous subjects, different methodological approaches had been dealt with in PE in a transversal way. These subjects are called “Educational Game in PE” “Inclusive PE” and “Sports Initiation.” The main idea is that future teachers acquire the necessary knowledge to be able to apply the model with guarantees within the classroom in the near future. This was verified in their school practice phase carried out in the third and fourth grades. These practices were developed in 8 public schools in the city of Burgos (Spain). The choice of these 13 participants was intended to meet a double criterion: (a) the participants’ voluntariness; (b) the best academic record in the subject of pedagogical models in PE. These criteria guarantee the correct acquisition of the CL by the participants, which ensures greater reliability of the results. The students with the best grades proved to have a better understanding of the essence of the CL, which ensured better replicability of the model in schools. Three schools where the internships took place were subsidized, while the rest were publicly owned. Two schools were in municipalities near the capital, while the others were in the city itself. None of them had more than 600 students. There was no substantial difference in the characteristics of the student in all of the schools, except for two schools located in a neighborhood with a lower socio-economic level. This did not decisively influence the application of the CL by the future teachers. In none of the schools was the CL being applied as a PE methodology, which eliminated the possible bias of the interventions. These interventions were carried out with students from all grades of the primary stage.

The training given on CL was carried out by a 34 years old teacher, doctor, with 10 years of experience in the university field in the same degree and university, and specialist in the teaching of school PE.

### Instruments

Three different instruments were used for the collection of information: interviews, teaching diaries and the seminars with the teacher. The questions in each instrument are clearly connected to the objectives of the study and to the categories that structure the results, thus giving reliability to the study (Swaminathan and Mulvihill, 2018). These tools make it possible to gather in a detailed and in-depth way all the information concerning the experiences of future EP teachers on the implementation of the CL. In addition, the use of Teachers’ Diaries and Seminars with the professor allowed for reflection throughout the intervention, redirecting professional practice according to the problems encountered. Teachers’ Diaries have proven to be an ideal qualitative tool for building the professional identity of novice teachers (Gut et al., 2016).

*Interviews*: These were carried out individually with the participants at the end of the intervention and were of a semi-structured nature (Table 1). Issues related to the initial expectations generated, to the results obtained after the implementation of the CL in the classroom and to the main benefits and problems found for its future replicability were addressed.

**TABLE 1:** Basic script used for semi-structured interviews with participants.

• What were your initial intentions before you carried out the process? • How did you organize the work in the classroom based on cooperative learning? • What has the application of the CL in the classroom brought you? • What have been the main problems you have encountered? • What would you change if you had to make a new intervention with the CL? • Will you continue applying the CL in your professional future? Why?

*Teachers’ diaries*: each participant made his or her own. They had a structured character based on the categories of analysis of the results: (a) initial expectations in the application of the CL; (b) problems encountered in its implementation; (c) reflection on its application in the future. The participants were writing in the same weekly throughout the 12 weeks that the intervention lasted. It is a tool that allows the teacher to take a perspective on the development of his/her own practice, and that, applied over time, favors a greater understanding of the context in which one acts (Safronov et al., 2020).

*Seminars with the professor*: were carried out throughout the intervention. Before the beginning of the application in the classroom, the professor who taught them about C established with the participants four seminars, spaced approximately every three weeks. They were conducted together, in order to exchange opinions and experiences about the results that were being found in each of the contexts. It is a clearly reflective instrument of great utility within the educational action-research process (Liu and Wang, 2018).

### Design and Procedure

The research has been structured in four distinct phases throughout the 2018–2019 school year:

*Phase 1. Structuring of the study and establishment of the schedule*: the study arose from the need to verify to what extent the initial training received by PE teachers on CL was subsequently replicated in the classroom. The usefulness and contribution of the research was confirmed since, through a qualitative approach, the aim was to check the experiences of future teachers in their practice contexts.

*Phase 2. Contact with the participants and delimitation of the CL intervention in the classroom*: having taught about CL to all the participants, it was easy to contact them to carry out the research. They were summoned before the start of their 12-week internship in order to record data from the schools they were going to work in. In addition, the intervention to be carried out was specified to be homogeneous in all cases. The intervention of all participants was based on three basic principles: (a) to respect the five elements that make up the CL; (b) to eliminate competitive proposals within the classroom; (c) to carry out the same didactic units in the intervention. The choice of these criteria was made in view of the importance of respecting the structure that gives the CL its essence, thus giving it sufficient rigor in its application (Fernández-Río et al., 2018). In this way, it was guaranteed that any possibility of competition would be eliminated. It was agreed to carry out the pedagogical interventions through the same contents, in order to maintain the fidelity in the totality of the interventions. These units were three. The first one was about games and cooperative activities, where a series of group challenges were posed to be solved in a motor way. These activities were related to transporting objects, overcoming obstacles, passing and receiving mobile elements and trusting one’s peers. The second unit of work was body expression, specifically shadow theater, where groups worked with a light bulb and a white cloth to make different shadows and create a narrative story. The third didactic unit focused on the work of motor skills and physical condition, where the four basic physical capacities were worked on through cooperative team challenges, thus obtaining rewards to overcome different levels. The aim of this program was to implement CL through a diversity of contents, demonstrating in a practical way how the phases and elements that structure the model were applied. This training was related to the previous learning that the students had in relation to other models, deriving in the possibility of its hybridization. The characteristics of the intervention are based on reflection, action research and the replicability of learning. The theory was clearly linked to practice, establishing constant reflections on what happened in each of the activities. This would allow them to act with greater rigor when they were in the schools.

Before carrying out the field work, the teacher prepared a script with the timing of the sessions to be carried out by the participants. This made it possible to guarantee a reliable and homogeneous intervention. Throughout the intervention in the schools it was corroborated that this script was respected.

*Phase 3. Carrying out of the seminars with the participants*: these seminars were carried out throughout the 12 weeks of intervention. A total of four were held. A seminar was established every three weeks. This was considered to be an adequate time, maintaining the balance between autonomous classroom practice of future teachers and the need for advice. All the participants were summoned to a seminar at the university in order to dialogue, share, advise and guide their interventions. The teacher used a script, divided into four parts, to maintain the monitoring structure: (a) compliance with the established plan; (b) type of problems encountered in the classroom; (c) how to solve these problems; (d) comments, questions and suggestions. In these seminars, in rotational order, each participant presented the main problems they had encountered in the classroom, in order to find common patterns of action that could be useful to all. Subsequently, decisions were made to try to reverse these problems found in the classroom. Each seminar lasted approximately 2 h. All were recorded on audio, for later listening when recapitulating the data.

*Phase 4. Conducting the interviews and data analysis*: at the end of the intervention, individual interviews were conducted with each of the participants. These interviews were carried out in the teacher’s office. Each one had a duration of 45 min and they were recorded in audio. In this last phase the teacher also received the teachers’ diary. From here, all the data obtained was transcribed and dumped into the text analysis computer program and the data was analyzed. In addition, the researchers reflected deeply on the purpose of the study, the procedure carried out and its adequacy to the objective set.

### Data Analysis

A qualitative approach was used to gain an in-depth understanding of how future teachers were able to apply the CL learned at university in the classroom. To do so, it was essential to know in first person the perception of the participants throughout the process, verifying how they were gradually transforming their school contexts and creating their professional identity. The fact that the main source of data was the assessments and experiences of those involved in the process allowed us to approach the study phenomenon in a real and interpretative way (Rubel and Okech, 2017). A triangulation was carried out between the information obtained in the data collection instruments with the aim of guaranteeing the reliability, transferability and credibility of the results. In addition, the most significant text extracts were coded in each of the instruments, using cross matching patterns (Saldaña, 2009). The researchers took an active part in the field work, reflecting throughout the process on the linearity of the results with the objectives of the study. The information was articulated, grouping it by thematic axes in the categories generated by means of a selective, open and axial codification (Strauss and Corbin, 2002).

### Generation of Categories and Their Categorization

Once the data from each instrument used was transcribed, it was dumped into the WEFT QDA computer and analysis program. Through the saturation of coinciding texts and ideas and the treatment of thematic axes, the information was grouped into the three initial categories of the study: (a) initial expectations in the application of the CL; (b) problems encountered in its implementation; (c) reflection on its application in the future. These categories are common to all the data collection instruments used, and are used to structure the analysis of the results. They are related to the object of study and the design of research, thus respecting the criteria of specificity and coherence that all qualitative research must have (Le Roux, 2017).

*Initial expectations in the application of the CL*: aspects related to the way of understanding and applying CL in the classroom, its educational purposes and the factors that directly influence its school treatment are addressed.

*Problems encountered in its implementation*: information regarding the problems encountered when applying the CL in the classroom and the guidelines and orientations established to successfully redirect the process are analyzed.

*Reflection about its application in the future*: the data linked to which are the positions and attitudes that future FE teachers have in relation to the application of the CL in the future are integrated, taking into account what happened after their first experience.

### Coding of Data Collection Instruments

Different acronyms are used to identify the text extracts with the data collection instrument from which they come. In relation to the interviews, it is used (EP1, EP2, EP3…) depending on the participant interviewed. Likewise, with regard to the teaching diaries, it is used (DD1, DD2, DD3…). For the seminars with the teacher, the acronym (SP1, SP2, SP3, and SP4) is used, taking into account the four seminars held throughout the intervention.

## Results

With regard to data coding, the following primary and secondary issues were obtained:

Initial expectations in the application of the CA: The primary issues were the understanding and application of the CL, while the secondary issues were the expectations, fears and uncertainty generated about the functioning of the model.

Problems encountered in its implementation: The primary issues were the excessive competitiveness of the students and their lack of experience, while the secondary issues were the lack of motor commitment, and the use of adequate space and materials.

Reflection on its future application: The primary was the need for further training in the model, while the secondary was the importance of reflection throughout the process and confidence in the model’s potential. All the information extracted from the three data collection instruments is grouped into the three categories of the study. Through the analysis of crossed patterns, the number of resulting literal text extracts is presented, showing the most significant and coinciding ones. Presenting the most consistent text extracts in each of the categories, in relation to the objectives of the study, favors the elimination of possible biases on the part of the researchers and guarantees the reliability of qualitative studies (Peterson, 2019). The qualitative methodology must have its main strength in the analysis of the participants’ experiences through the text, being not at all less rigorous than the quantitative one (Wallace and Kuo, 2020). In this case, the results follow a research structure linked to four of the five qualitative approaches (Creswell, 2007; Creswell et al., 2007): case study, narrative, phenomenology and action research.

### Initial Expectations in the Application of the CA (302 Text Extracts)

It can be seen how the expectations that the future teachers had at the beginning were not completely satisfied, finding problems in practice such as student boredom and lack of control over the classroom:

“I honestly thought that the students would respond very well to cooperative learning activities, however, many of them said that they were bored and wanted to do something else” (EP5). “I go home frustrated, because I had prepared the session very well, I thought it would be fun for them, but I spent the whole class trying to motivate them towards the activities […] (DD9).” “It was a direct hit with reality. From the beginning I lost control of the class, and I was more concerned about being attended and listened to than actually doing the activities. The students disconnected as soon as the explanations were a little long” (SP1).

It also reflects how future teachers thought they knew the CL model, although later in the classroom many doubts and uncertainties were generated when applying it in terms of aspects such as group management, application of content and time management:

“I thought I was clear about the principles of the CL, and how through them the class was going to cooperate. However, from the first day I realized that it was not going to be easy, as I found it difficult to adapt the activities to the characteristics of the groups” (EP10). “In principle I thought that the application of the CL could be carried out through any content, but for example in those of a sporting nature I find it difficult to eliminate competition” (DD5). “One of the main ones for me is managing class time. I think that in one class I will be able to do three or four activities, when at most I have time for two” (SP2).

Participants highlight how the reality of the classroom with children differs greatly from what was learned and experienced at the university:

“When they tell you about the benefits of CL in the university you only see advantages, everything seems very nice and you think it will be easy to apply” (EP1). “They show you videos of the kids doing activities and you are shocked […]. You can’t wait to get to the classroom to try them out” (EP6). “When you get to class everything changes, you realize that everything in college was too idyllic” (DD9). “Among us adults, there was silence, more autonomy and when something was proposed everyone did it without a problem […] When you put it to the children it is a totally different reality, more complex” (SP3).

### Problems Encountered in Their Implementation (318 Text Extracts)

One of the main problems perceived by future teachers was the excessive competition among students:

“Children are very used to competing in PE, and this is noticeable from day one” (EP7). “They always want to win above all else and this makes it very difficult for them to enter into the logic of cooperation” (EP12). “Whenever I propose a cooperative activity there are always a couple of students in class who want to solve it by themselves as soon as possible, and they also tend to make fun of those who are not capable” (DD13). “They are so used to winning and losing that when they have to think as a team there are some who tend to get bored […] There is also a group of students who do not talk for fear of being laughed at” (DD6). “Some class members are not able to respect each other’s waiting times and different learning rhythms… they think more individually than collectively and this is hard to change” (SP4).

It was also observed how future teachers perceived greater ease in applying the model to those contents more linked to cooperative activities than to those related to physical condition:

“When you are doing team activities it is much easier to apply the model, as the guidelines are much clearer” (EP9). “I realized how in physical fitness activities we act more competitively and it becomes more difficult.” (DD10). “In some content related to games I usually go to win individually, so implementing the model sometimes becomes more complicated” (DD5). “The main challenge is to be able to apply the cooperative learning model above any content” (SP2).

Another problem expressed by the future teachers was the lack of motor commitment involved in the implementation of the CL:

“I am aware that the CL involves reflection and dialogue among participants […]. The problem is when this time is too long, which causes the time of motor practice to be reduced” (EP4). “You can see how some children disconnect when the explanation is too long” (EP8). “On the one hand I know that the methodology has these characteristics, but on the other hand I think that from the PE we should encourage movement and the practice of physical activity, so I am faced with an eternal dilemma” (DD12). “It is the students themselves who often say that they are bored, commenting that when we practice a sport […] Many times you think that what you are doing makes no sense and does not motivate them. This makes me very angry, since I prepare my classes a lot” (SP3).

### Reflection on Its Future Application (324 Text Extracts)

The results of this variable indicate how future teachers express the need for further training in the model:

“This classroom experience has helped me realize what I still need to learn from cooperative learning” (EP10) “I thought I already knew a lot about cooperative learning, but reality has shown me that I still have to justify the tasks much better, their adaptation to the students, to the space […]” (EE8) “Some things are not going well for me, but the most important thing is that I am aware of why, so I will be able to change it the next times I develop these tasks in class.” (DD7) “This is to be taken as an apprenticeship in our training […]. We must not despair, it is our first experience in the classroom, and we still have a long way to go in our formation” (SP2).

They also highlighted the satisfaction and importance of the seminars held with the professor throughout the intervention:

“The seminars have helped me to know that I was not the only person who had problems, and that things were happening to the rest of my classmates in class that were very similar to mine” (EE2). “We have always been told at the university about the importance of teaching reflection on practice, and the seminars we have done have helped me to realize this” (DD13) “I was looking forward to this seminar to share with you everything that is happening to me and see how the intervention is going […]. It is an ideal way to share and look for common strategies” (SS3).

Although the participants have reflected a diversity of problems in the implementation of the model in the classroom, they remain confident in their potential and in continuing to apply it in their professional future:

“Having had certain problems at the beginning is normal, but I still believe that this model is fundamental in contributing to the type of physical education that we want” (EE1) “Problems in the classroom are normal with this methodology and with any other […] Mistakes serve to continue learning” (EE4) “Although I have had problems, there have been more positive than negative things, and I honestly want to continue applying this methodology in my future” (DD8) “If you believe in participatory physical education, you must use models such as the cooperative model, otherwise the principles behind the subject are totally different” (SS4).

## Discussion

The aim of the study was to analyze to what extent future PE teachers were able to apply the CL training they have received at university in the classroom, by exploring their fears, insecurities and problems in doing so. The results show how the initial expectations that future PE teachers had are not matched in reality, finding a variety of problems when applying the C model in the classroom. However, they show that they understand the difficulty involved and intend to continue applying it in their professional future. The results obtained connect perfectly with the theory of occupational socialization (Richards et al., 2014), which tries to connect the initial training of teachers with the professional reality they will find in the classroom. To do so, it is essential to pay attention to the pedagogy of dialogue, actively following up on the future teachers in their first contacts with the classroom. This will allow them to achieve confidence and competence in themselves to create their own professional identity (Eather et al., 2019).

In relation to the first category of the study, related to the initial expectations, the reality in the school showed them the complexity that has to apply the CL with the children, manifesting how different everything is in relation to what was experienced in the university. This is one of the main problems that exist today in the initial training of teachers, where they are trained more on theoretical aspects than on the acquisition of practical resources for application in the classroom (Richards et al., 2013). From the perspective of pedagogical coherence, university teachers must maintain a close relationship with what is happening in the schools, showing scientific evidence of which methods, strategies and protocols are the best to follow in each context. As indicated by Greaves et al. (2019), the university must provide channels so that future teachers can apply what they have learned in their subjects, with the possibility of experimentation and above all with guarantees of redirecting teaching processes. The data from the study have reflected the perceived shortcomings in managing groups and class time well according to the content selected. It is common for new teachers to fall into the error of “more is better,” considering that many activities are necessary in order to generate more learning. In fact, the CL is characterized by a high component of group reflection, which means that in many cases the number of activities is not the most relevant element in the teacher’s planning (Walker and Johnson, 2018). On the other hand, the participants in the study have reflected the frustration they felt when students were sometimes bored in class. This is common when students first receive the CL in PE, as they are usually more accustomed to traditional PE models in which physiological activation takes precedence over social and learning aspects. Dyson et al. (2016) indicate that despite the initial resistance that we may encounter in schools, students and even families when applying the CL, we must be consistent in its application until the student acts under the logic of cooperation. This full conviction can serve as a stimulus for the professional development of teachers, thus increasing the possibilities of applying the pedagogical model correctly.

With regard to the second category of the study concerning the problems encountered, two were the main axes found. On the one hand, the excessive competitiveness of students, and on the other, the perceived lack of motor commitment. It is usual that the activities applied in the PE are based on the immediacy of the objective to be achieved from an individual approach (throwing faster, arriving running before the colleagues, making more jumps than the others…). This type of conception of the subject, applied year after year, creates in the student a driving culture, where what matters is individuality above any collective, creative and social criteria (Pill and SueSee, 2017). When this logic of acting is imposed, the students with more individual success reject any kind of change in the subject, since the protagonism to which they were accustomed is reduced. This is why new teachers must be guided and accompanied throughout these training processes, since they produce great contrasts with respect to their initial pretensions (Richards and Templin, 2011). In this sense, even today the “wet T-shirt syndrome” still prevails within the PE classes, understanding that if the student does not sweat in the PE classes is not generating real learning. However, several studies (Hortigüela-Alcalá et al., 2017, 2018) indicate that the motivation and predisposition of PE teachers to teach one content or another is not linked to the intensity of the level of physical practice required by the content. Therefore, teaching programs must be aimed at reducing the competitiveness of students, designing protocols to monitor each of the strategies implemented (Johnson and Johnson, 2009). Connecting with the ideas addressed in the theoretical framework, the results have shown the various problems encountered by future PE teachers. This is why CL must be taught in universities as an in-depth pedagogical model, and not in isolation at specific moments. Its possibilities are infinite, but if it is not properly focused, it can have the opposite effect to that intended at the beginning, for example in the dialogue that it requires among students for learning sports (Darnis and Lafont, 2015). It is essential to have clear teaching structures that can be applied transversely to any content, with the objective of fostering social relations through motor skills (O’Leary et al., 2015).

The last category of the study has been about the reflection of the future PE teachers to apply the CL in their professional future. Firstly, the future teachers express the need to continue being trained in the model, assuming its complexity and considering the experience a key factor to be able to continue evolving as professionals. In this sense, Bores-García et al. (2020) highlight the importance of the activities carried out in the classroom being governed by the principles that give meaning to the CL model, and for this, teachers must ensure that these activities can be extrapolated to a variety of contexts. In this sense, the participants of the study highlighted as very relevant and necessary the seminars that were held with the teacher throughout the intervention, since they allowed them to reconstruct their steps in the school and to reflect on the problems that were encountered throughout the process. Lynch and Curtner-Smith (2019) criticize the poor connection that in many cases exists between education faculties and schools, explaining how an education system is broken if the predominant socio-cultural influences in each context are not intentionally addressed. There is still a large gap between initial teacher training and the reality of schools, which creates a tension between theory and practice. In order to reduce this tension, it is essential that teacher trainers develop auto-ethnographic processes in order to understand the applicability of learning (Yung, 2020). Some authors advocate the need for an intense reformulation of initial teacher training, establishing innovative training plans that adapt to an increasingly changing educational reality (Yeigh and Lynch, 2017). Finally, the results show how future teachers, after the experience, continue to trust in the potential derived from the CL, hoping to continue applying it in their professional future. This is why, from the initial teacher training, the development of practices in real contexts must be guaranteed, thus contributing to the generation of the construction of a clear professional identity that can be transferred to their daily life at school (Androusou and Tsafos, 2018). In the present investigation, a 12-week intervention has been carried out, addressing the entire duration of the course. It is ideal to be able to establish interventions of the longest possible duration, thus guaranteeing the establishment of significant learning (Watts et al., 2019).

## Conclusion

In relation to the objective of the study, it has been verified how the future teachers of PE have not seen their initial expectations fulfilled in the application of the CL in the school, finding diverse problems like the management of the time, the motivation of the student or the scarce motor commitment of the activities. However, they consider the experience to be very positive, hoping to continue applying the model in their professional future. The main contribution of this research, besides generating new evidences about the applicability of the CL, has been the use of a contrast of qualitative instruments that allow to reflect and redirect the practices of the future PE teachers. In addition, seminars were carried out with the participants throughout the intervention process, thus contributing to the pedagogy of dialogue and the theory of occupational socialization. This is a contribution to the previous literature, since it allows for the establishment of connections between the initial training of PE teachers and schools, through designs based on reflection and the construction of professional identity.

However, the article has some limitations. On the one hand, only one intervention is measured. On the other hand, only the assessments experienced by future PE teachers are taken into account. Therefore, it would be interesting to contrast these results with future interventions of a longitudinal nature, thus verifying the level of evolution of the participants with respect to the application of the CL. In this sense, it would also be possible to attend to the students’ assessments. In addition, it would be interesting to make interventions by increasing the number of participants, as well as the establishment of teaching programs based on a diversity of research.

We consider this research of special interest for all those PE teachers who work the CL in the school. Also for all those responsible for the elaboration of school curricula or for the management of physical activity programs, understanding that the cooperation has to be closely linked to the sport field. It is necessary to continue investigating in this line and throwing light on the bonanzas and guarantees of the application of the CL in PE.

## Data Availability Statement

All datasets generated for this study are included in the article/supplementary material.

## Ethics Statement

The studies involving human participants were reviewed and approved by the Bioethics Committee of the University of Burgos. The participants provided written informed consent to participate in this study.

## Author Contributions

DH-A: conceptualization, validation, and investigation. AH-G: investigation and visualization. SG-V: investigation and formal analysis. JP-V: investigation and resources. AB-E: review and editing. All authors contributed to the article and approved the submitted version.

## Conflict of Interest

The authors declare that the research was conducted in the absence of any commercial or financial relationships that could be construed as a potential conflict of interest.
